# Supplementary material for: Genomic Surveillance Uncovers a 10-Year Persistence of an OXA-24/40 Acinetobacter baumannii Clone in a Tertiary Hospital in Northern Spain
Source: Int J Mol Sci. 2024 Feb 16;25(4):2333. doi: 10.3390/ijms25042333 (PMC10889530; doi:10.3390/ijms25042333)
Supplement: Supplementary file 1 [file ijms-25-02333-s001.zip › ijms-2833003-supplementary.pdf]

Supplementary Material

Table S1. Characteristics of clinical isolates and control strains included in this study. * Environmental isolate. HUC: Hospital Universitario Cruces; N/A: Not Applicable.

Isolate	Collection Date	Patient gender	Patient age (years)	Clinical sample	Hospital	Ward	Clinical features	*Genbank Accession number*
HUC-SM28 (Control)	26/04/1999	Male	82	Sputum	HUC	IM for Elder Care	Pneumonia	SAMN39242203
HUC-A11 (Control)	25/02/2002	Male	76	NA	HUC	IM for Elder Care	Bronchiectasia	SAMN39242202
HUC-86	18/07/2009	Male	25	Blood culture	HUC	Recovery Unit	Bacteremia	SAMN39242188
HUC-88	17/02/2009	Male	40	Sputum	HUC	Recovery Unit	Pneumonia	SAMN39242189
HUC-89	03/06/2009	Male	61	Swab	HUC	Major Burns Unit	Third-degree burn	SAMN39242190
HUC-90	26/06/2009	Male	49	Surveillance swab	HUC	ICU	Aortic aneurysm	SAMN39242191
HUC-92	18/09/2009	Male	50	Surveillance swab	HUC	ID Dept	Multiple sclerosis	SAMN39242192
HUC-93	13/08/2009	Male	85	Skin swab	HUC	IM for Elder Care	CVA (Cerebral Vascular Accident)	SAMN39242193
HUC-94	12/08/2009	Male	49	Surveillance swab	HUC	ICU	Tetraparesis	SAMN39242194
HUC-96	02/07/2009	Male	49	Swab	HUC	ICU	Poliraduculitis	SAMN39242195
HUC-97	17/06/2009	Male	50	Swab	HUC	ICU	Pneumonia	SAMN39242196
HUC-98	26/06/2009	Male	51	Surveillance swab	HUC	ICU	Brain aneurysm	SAMN39242197
HUC-99	25/06/2009	Male	40	Not surgical wound	HUC	Internal Medicine	Brain aneurysm	SAMN39242198
HUC-100	24/08/2009	Female	67	Tip of Intravascular catheter	HUC	Major Burns Unit	Third-degree burn	SAMN39242199
HUC-101	24/08/2009	Male	47	Tip of Intravascular catheter	HUC	ICU	Bacteraemia	SAMN39243021
HUC-106	09/10/2009	Male	48	Swab	HUC	Recovery Unit	Quadriplegia	SAMN39242200
HUC-109	22/06/2009	Female	36	Tip of Intravascular catheter	HUC	ICU	Cushing´s syndrome dysregulation	SAMN39242201

Hi873	07/12/2019	Female	32	Inguinal swab (burn)	HUC	Major Burns Unit	Third-degree burn	SAMN39242200
Aba1516	11/06/2020	Male	64	Surveillance swab	HUC	ICU	COVID19	SAMN39242205
Aba1517	11/06/2020	Female	53	Blood culture	HUC	ICU	Bacteraemia	SAMN39242206
Aba1518	15/06/2020	Male	73	Inguinal swab	HUC	ICU	COVID19	SAMN39242207
Aba1519	02/06/2020	Female	63	Urinary catheterization	HUC	ICU	Quadriplegia	SAMN39242208
*Aba1520**	15/06/2020	N/A	N/A	Patient room 1: bedrails	HUC	ICU	N/A	SAMN39242209
Aba1521	14/07/2020	Male	72	Surgical wound	HUC	ICU	COVID19	SAMN39242210
Aba1522	13/10/2020	Male	64	Blood culture	HUC	ICU	COVID19	SAMN39242211
Aba1523	13/10/2020	Male	43	Rectal swab	HUC	ICU	COVID19	SAMN39242212
Aba1524	12/10/2020	Male	64	Blood culture	HUC	ICU	Bacteraemia	SAMN39242213
Aba1525	27/09/2020	Female	47	Pharyngeal swab	HUC	ICU	Kidney transplant, hepatic pathology, COVID19	SAMN39242214
Aba1526	20/10/2020	Male	62	Inguinal swab	HUC	ICU	Kidney transplant failure	SAMN39242215
Aba1527	24/10/2020	Female	35	Blood culture	HUC	ICU	Bacteraemia	SAMN39242216
Aba1528	17/07/2020	Female	65	Surgical wound	HUC	ICU	Cardiopathy	SAMN39242217
*Aba1529**	16/10/2020	N/A	N/A	Patient room 2: furniture	HUC	ICU	N/A	SAMN39242218
*Aba1530**	16/10/2020	N/A	N/A	Patient room 2: bedrails	HUC	ICU	N/A	SAMN39242219
*Aba1531**	30/09/2020	N/A	N/A	Patient room 3: mattress	HUC	ICU	N/A	SAMN39242220
*Aba1532**	24/08/2020	N/A	N/A	Patient room 4: bedrails	HUC	ICU	N/A	SAMN39242221
*Aba1533**	23/10/2020	N/A	N/A	Patient room 4: bedrails	HUC	ICU	N/A	SAMN39242222
*Aba1534**	23/10/2020	N/A	N/A	Patient room 5: mattress	HUC	ICU	N/A	SAMN39242223
Aba1552	04/01/2021	Male	64	Urine	HUC	ICU	COVID19	SAMN39242224

Table S2. Antibiotic susceptibility profile of the CRAb isolates belonging to Outbreak 1 and control strains.

	MIC (mg/L)										
**Isolate**	**Imipenem**	**Meropenem**	**Gentamicin**	**Tobramycin**	**Amikacin**	**Ciprofloxacin**	**Levofloxacin**	**Cotrimoxazole**	**Minocycline***	**Tigecycline****	**Colistin**
HUC-86	>=16, R	>=16, R	>=16, R	>=16, R	8, S***	>=4, R	>=8, R	>4/76, R	<=1	4, R	<=0,5, S
HUC-88	>=16, R	>=16, R	>=16, R	8, R	32, R	>=4, R	>=8, R	>4/76, R	<=1	4, R	>=16, R
HUC-89	>=16, R	>=16, R	>=16, R	8, R	16, R	>=4, R	>=8, R	>4/76, R	<=1	4, R	<=0,5, S
HUC-90	>=16, R	>=16, R	>=16, R	>=16, R	32, R	>=4, R	>=8, R	>4/76, R	<=1	4, R	<=0,5, S
HUC-92	>=16, R	>=16, R	>=16, R	8, R	8, S***	>=4, R	>=8, R	>4/76, R	<=1	4, R	<=0,5, S
HUC-93	>=16, R	>=16, R	>=16, R	8, R	8, S***	>=4, R	>=8, R	>4/76, R	<=1	1, R	<=0,5, S
HUC-94	>=16, R	>=16, R	>=16, R	8, R	16, R	>=4, R	>=8, R	>4/76, R	<=1	4, R	<=0,5, S
HUC-96	>=16, R	>=16, R	>=16, R	>=16, R	32, R	>=4, R	>=8, R	>4/76, R	<=1	4, R	<=0,5, S
HUC-97	>=16, R	>=16, R	>=16, R	8, R	32, R	>=4, R	>=8, R	>4/76, R	<=1	4, R	<=0,5, S
HUC-98	>=16, R	>=16, R	>=16, R	>=16, R	32, R	>=4, R	>=8, R	>4/76, R	<=1	4, R	<=0,5, S
HUC-99	>=16, R	>=16, R	>=16, R	>=16, R	>=64, R	>=4, R	>=8, R	>4/76, R	<=1	4, R	<=0,5, S
HUC-100	>=16, R	>=16, R	>=16, R	>=16, R	16, R	>=4, R	>=8, R	>4/76, R	<=1	2, R	<=0,5, S
HUC-101	>=16, R	>=16, R	>=16, R	>=16, R	16, R	>=4, R	>=8, R	>4/76, R	<=1	2, R	<=0,5, S
HUC-106	>=16, R	>=16, R	8, R	4, S***	<=2, S***	>=4, R	>=8, R	2/38, S	<=1	<=0,5, S	<=0,5, S
HUC-109	>=16, R	>=16, R	8, R	4, S***	<=2, S***	>=4, R	>=8, R	2/38, S	<=1	<=0,5, S	<=0,5, S
HUC-A11	>=16, R	>=16, R	>=16, R	>=16, R	>=64, R	>=4, R	>=8, R	>4/76, R	<=1	1, R	<=0,5, S
HUC-SM28	>=16, R	>=16, R	>=16, R	>=16, R	>=64, R	>=4, R	>=8, R	>4/76, R	<=1	4, R	<=0,5, S
MIC_{50} (mg/L)	>=16, R	>=16, R	>=16, R	>=16, R	32, R	>=4, R	>=8, R	>4/76, R	<=1	4, R	<=0,5, S
MIC_{90} (mg/L)	>=16, R	>=16, R	>=16, R	>=16, R	>=64, R	>=4, R	>=8, R	>4/76, R	<=1	4, R	<=0,5, S

* There are no breakpoints available as EUCAST considers the agent is unsuitable for treatment of systemic infections

** In the absence of EUCAST breakpoints for this organism, breakpoint of 0.5 mg/L for Enterobacterales was used

*** According to EUCAST there is insufficient evidence to recommend this agent in monotherapy

Table S3. Antibiotic susceptibility profile of the CRAb isolates belonging to Outbreak 2.

Isolate	MIC (mg/L)										
	**Imipenem**	**Meropenem**	**Gentamicin**	**Tobramycin**	**Amikacin**	**Ciprofloxacin**	**Levofloxacin**	**Cotrimoxazole**	**Minocycline***	**Tigecycline****	**Colistin**
Hi873	>8, R	>8, R	>8, R	>8, R	32, R	>2, R	>4, R	>4/76, R	<=1	2, R	<=0,5, S
Aba1516	>8, R	>8, R	>8, R	>8, R	32, R	>2, R	>4, R	>4/76, R	<=1	4, R	<=0,5, S
Aba1517	>8, R	>8, R	>8, R	>8, R	32, R	>2, R	>4, R	>4/76, R	<=1	4, R	<=0,5, S
Aba1518	>8, R	>8, R	>8, R	>8, R	32, R	>2, R	>4, R	>4/76, R	<=1	4, R	<=0,5, S
Aba1519	>8, R	>8, R	>8, R	>8, R	32, R	>2, R	>4, R	>4/76, R	<=1	4, R	<=0,5, S
Aba1520	>8, R	>8, R	>8, R	>8, R	32, R	>2, R	>4, R	>4/76, R	<=1	4, R	<=0,5, S
Aba1521	>8, R	>8, R	>8, R	>8, R	32, R	>2, R	>4, R	>4/76, R	<=1	4, R	<=0,5, S
Aba1522	>8, R	>8, R	>8, R	>8, R	32, R	>2, R	>4, R	>4/76, R	<=1	1, R	<=0,5, S
Aba1523	>8, R	>8, R	>8, R	>8, R	32, R	>2, R	>4, R	>4/76, R	<=1	1, R	<=0,5, S
Aba1524	>8, R	>8, R	>8, R	>8, R	32, R	>2, R	>4, R	>4/76, R	<=1	1, R	<=0,5, S
Aba1525	>8, R	>8, R	>8, R	>8, R	32, R	>2, R	>4, R	>4/76, R	<=1	1, R	<=0,5, S
Aba1526	>8, R	>8, R	>8, R	>8, R	32, R	>2, R	>4, R	>4/76, R	<=1	1, R	<=0,5, S
Aba1527	>8, R	>8, R	>8, R	>8, R	32, R	>2, R	>4, R	>4/76, R	<=1	4, R	<=0,5, S
Aba1528	>8, R	>8, R	>8, R	>8, R	32, R	>2, R	>4, R	>4/76, R	<=1	1, R	<=0,5, S
Aba1529	>8, R	>8, R	>8, R	>8, R	32, R	>2, R	>4, R	>4/76, R	<=1	1, R	<=0,5, S
Aba1530	>8, R	>8, R	>8, R	>8, R	32, R	>2, R	>4, R	>4/76, R	<=1	1, R	<=0,5, S
Aba1531	>8, R	>8, R	>8, R	>8, R	32, R	>2, R	>4, R	>4/76, R	<=1	1, R	<=0,5, S
Aba1532	>8, R	>8, R	>8, R	>8, R	32, R	>2, R	>4, R	>4/76, R	<=1	1, R	<=0,5, S
Aba1533	>8, R	>8, R	>8, R	>8, R	32, R	>2, R	>4, R	>4/76, R	<=1	1, R	<=0,5, S
Aba1534	>8, R	>8, R	>8, R	>8, R	32, R	>2, R	>4, R	>4/76, R	<=1	1, R	<=0,5, S
Aba1552	>8, R	>8, R	>8, R	>8, R	32, R	>2, R	>4, R	>4/76, R	<=1	1, R	<=0,5, S
MIC_{50} (mg/L)	>8, R	>8, R	>8, R	>8, R	32, R	>2, R	>4, R	>4/76, R	<=1	1, R	<=0,5, S
MIC_{90} (mg/L)	>8, R	>8, R	>8, R	>8, R	32, R	>2, R	>4, R	>4/76, R	<=1	4, R	<=0,5, S

* There are no breakpoints available as EUCAST considers the agent is unsuitable for treatment of systemic infections

** In the absence of EUCAST breakpoints for this organism, breakpoint of 0.5 mg/L for Enterobacterales was used
